# Optimizing Human–Robot Teaming Performance through Q-Learning-Based Task Load Adjustment and Physiological Data Analysis

**DOI:** 10.3390/s24092817

**Published:** 2024-04-28

**Authors:** Soroush Korivand, Gustavo Galvani, Arash Ajoudani, Jiaqi Gong, Nader Jalili

**Affiliations:** 1 Department of Mechanical Engineering, Southern Methodist University, Dallas, TX 75205, USA; skorivand@smu.edu; 2 Department of Mechanical Engineering, The University of Alabama, Tuscaloosa, AL 35487, USA; gmartinsgalvani@crimson.ua.edu; 3 Human-Robot Interfaces and Physical Interaction Laboratory (HRI^2^), Istituto Italiano di Tecnologia, 16163 Genoa, Italy; arash.ajoudani@iit.it; 4 Department of Computer Science, The University of Alabama, Tuscaloosa, AL 35487, USA

**Keywords:** human–robot teaming, Q-learning, machine learning, performance prediction, performance maximization, task load, task engagement, physiological data

## Abstract

The transition to Industry 4.0 and 5.0 underscores the need for integrating humans into manufacturing processes, shifting the focus towards customization and personalization rather than traditional mass production. However, human performance during task execution may vary. To ensure high human–robot teaming (HRT) performance, it is crucial to predict performance without negatively affecting task execution. Therefore, to predict performance indirectly, significant factors affecting human performance, such as engagement and task load (i.e., amount of cognitive, physical, and/or sensory resources required to perform a particular task), must be considered. Hence, we propose a framework to predict and maximize the HRT performance. For the prediction of task performance during the development phase, our methodology employs features extracted from physiological data as inputs. The labels for these predictions—categorized as accurate performance or inaccurate performance due to high/low task load—are meticulously crafted using a combination of the NASA TLX questionnaire, records of human performance in quality control tasks, and the application of Q-Learning to derive task-specific weights for the task load indices. This structured approach enables the deployment of our model to exclusively rely on physiological data for predicting performance, thereby achieving an accuracy rate of 95.45% in forecasting HRT performance. To maintain optimized HRT performance, this study further introduces a method of dynamically adjusting the robot’s speed in the case of low performance. This strategic adjustment is designed to effectively balance the task load, thereby enhancing the efficiency of human–robot collaboration.

## 1. Introduction

**Figure 1:**
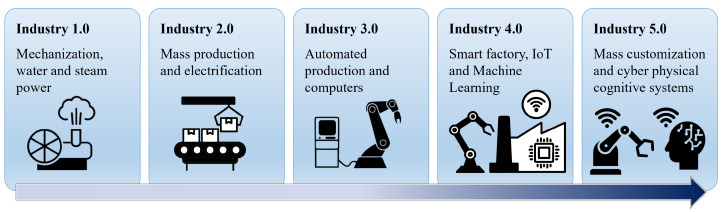
Evolution of industrial revolutions from Industry 1.0 to 5.0, showcasing the transition from mechanization to mass customization and the integration of human cognitive systems with robotics in modern manufacturing.

In traditional settings, robots have typically worked independently from humans. Even in settings where collaboration is potentially beneficial, such as in manufacturing, industrial robots have mostly operated in physically separated environments. This is due to safety and production design simplicity concerns [1,2,3]. Consequently, robots have been relegated to performing well-defined and repetitive tasks. However, as illustrated in Figure 1, the evolution towards Industry 5.0 is steering manufacturing from mass production to mass customization and personalization, leading to more complex and varied operational settings. To meet this demand, it is necessary to integrate humans into the manufacturing process, enabling the synergy of human critical thinking with robotic precision for tasks that are tedious, hazardous, or beyond human capabilities, such as operations at the nanoscale [4]. This underscores the growing significance of human–robot teaming (HRT) in the future of manufacturing [5,6,7].

In this research, we focus on a specific application within this evolving landscape: HRT for quality control (QC) tasks. In this configuration, a collaborative robot is programmed to deliver parts to a QC station where a human operator performs precision measurements using a caliper. These QC tasks are vital for maintaining product quality in manufacturing environments close to industrial machines that often lack advanced safety systems. Utilizing collaborative robots equipped with robust safety features significantly mitigates risks, enhancing both the safety and efficiency of the manufacturing process. This arrangement allows human operators to focus on accurate measurements, ensuring high-quality outcomes without the risks associated with high-speed industrial machinery.

**Figure 2:**
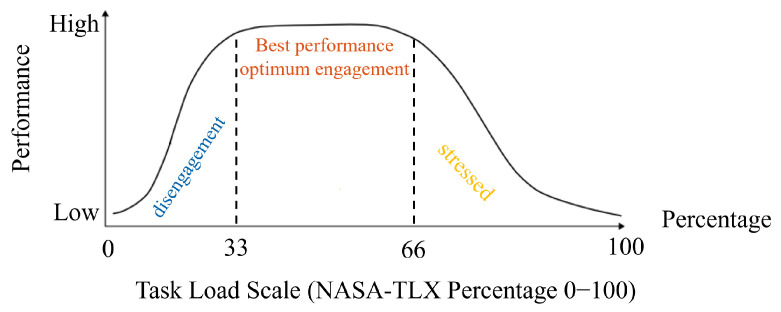
The relationship between task load and performance, with the task load scale derived from the NASA-TLX as a percentage. Performance varies with task load, where lower performance is associated with disengagement (task load < 33%), optimal performance corresponds to peak engagement (task load 33–66%), and higher task load leads to stress and reduced performance (task load > 66%) [1,8,10].

In dynamic and less structured environments, the variability of numerous parameters—including the internal state of human operators—can significantly influence the performance of HRT [1]. Managing task load and engagement levels is crucial for optimizing HRT performance. This management hinges on the Yerkes–Dodson Law, which posits that performance is optimal at a moderate level of arousal and deteriorates if arousal becomes too low or too high [8]. Figure 2 illustrates how, in adherence to this principle, our framework operationalizes the task load by dividing it into three distinct states—low, optimal, and high—each approximately one-third of the task load continuum [1]. This segmentation correlates with understimulation leading to disengagement, optimal arousal corresponding to peak performance, and overarousal resulting in stress or errors. Both disengagement and excessive stress can negatively impact accuracy, productivity, and multitasking capabilities, highlighting the need for a delicate balance to maintain team efficacy [9,10].

In this paper, task engagement within HRT for QC tasks is defined as the process through which operators initiate, maintain, and conclude their involvement with QC tasks. This engagement encompasses the execution of tasks in collaboration with the robot and is characterized by the operators’ sustained attention and commitment to achieving task objectives, alongside their capacity to adapt to and interact dynamically with the robot. Effective engagement is crucial, ensuring that operators remain focused on delivering high accuracy in measurements [11].

Similarly, task load is defined within the context of HRT for QC tasks as the operator’s subjective experience, influenced by the task’s objectives, the conditions under which the task is performed, and the interaction between the operator’s skills and perceptions. This experiential load includes mental and physical demands, temporal pressures, and subjective assessments of performance, effort, and frustration. It is measured through subjective evaluations that reflect the complex and dynamic nature of task load in collaborative environments with robots [12].

Furthermore, continuous exposure to stressful working conditions has been associated with an increased risk of work-related injuries and other health issues stemming from stress [13,14,15]. It is therefore critical to devise a predictive model capable of accurately estimating human performance within HRT settings. Such a model would enable adaptive decision-making and actions by both human and robotic agents, ensuring optimal collaboration. Implementing this model promises not only to uphold high levels of productivity but also to enhance the mental and physical health of human participants engaged in HRT operations.

To effectively predict and optimize HRT performance, focusing on variables with a significant impact is crucial, especially when direct measurement is unfeasible. Human engagement in task execution is a key variable significantly influencing performance [16,17]. High engagement and familiarity with a task typically correlate with enhanced performance. Conversely, disengagement or tasks perceived as overly challenging can lead to reduced performance [18]. Thus, continuously monitoring and accurately assessing a human’s level of engagement in a specific task is essential for predicting and maximizing performance in HRT.

Numerous factors contribute to an individual’s levels of engagement, disengagement, or stress during task execution. A pivotal element among these is the cognitive demand imposed by the task on the individual. Insufficient cognitive load can lead to task disengagement, whereas an excessive cognitive load may surpass the individual’s capacity, adversely affecting their performance [1,19]. Consequently, optimizing cognitive load is crucial for achieving optimal performance, sustaining engagement, and fostering healthy working conditions.

Temporal demand plays a pivotal role in influencing an individual’s engagement with a task [20,21]. In the context of HRT, discrepancies in pace between the human and the robot can lead to engagement issues. Specifically, if the human outpaces the robot, they might experience boredom and disengage. Conversely, if the individual struggles to keep up with the task’s pace, stress can ensue. Other factors, such as physical demand [22] and negative emotions [23], also significantly impact task engagement. The NASA TLX (National Aeronautics and Space Administration Task Load Index) is an established tool for evaluating these indices [24]. Task load encompasses both cognitive and physical efforts required for task completion, varying with task complexity, the allocated time, and the individual’s expertise.

The NASA TLX has proven to be a reliable and widely used method for assessing task load in various domains, including healthcare [25], manufacturing [26], and education [27]. This tool assesses task load based on six indices; however, the importance of each index varies significantly across different tasks, and assigning accurate weights to each index presents a complex challenge [28]. To tackle this, we implemented Q-Learning. This approach allows us to precisely calibrate the weights for each index, enhancing the reliability of our task load evaluations.

Reinforcement learning (RL) provides a robust framework for decision making in environments where explicit models are infeasible. Among various RL algorithms, Q-learning, a model-free and off-policy reinforcement learning algorithm, is particularly suited for scenarios where the sample size is small compared with the dimensions of the state space. Its robustness and its capability to learn optimal policies without prior knowledge of the environment’s dynamics significantly enhance its applicability to our study. Other RL methods, each with distinct characteristics and operational demands, offer complementary approaches that will be explored in future research to fully harness their strengths in complex interaction tasks.

In higher levels of HRT autonomy, where the robot adapts to the varying inner states of the human, relying solely on self-reported task load may not be reliable. Recent research indicates that human physiological signals offer a robust measure of cognitive and task loads [29,30,31], providing a more reliable indicator than self-report methods alone. These signals can be conveniently captured using wearable devices, such as wristbands, during task execution. Employing a range of machine learning (ML) techniques, our objective is to highlight the relationship between task load, performance, and their physiological responses. Additionally, the insights garnered from this study have implications that extend beyond HRT to sectors such as healthcare, aviation, and education, where understanding the weighted task load indices is crucial for optimizing performance and enhancing individual well-being.

**Figure 3:**
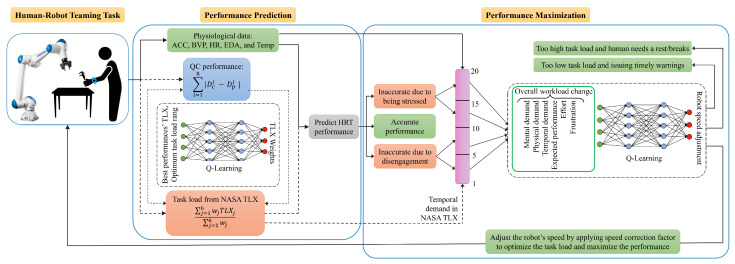
This diagram depicts our approach to predicting and improving HRT performance, starting with collecting physiological and QC measurement data, alongside task load information from NASA TLX questionnaire responses. A Q-Learning algorithm calculates task load weights to classify participants into three groups: those with accurate performance, those with stress-induced inaccurate performance, and those with disengagement-induced inaccurate performance. We then predict HRT performance and adjust it by changing the robot’s speed. If task load is too high for speed adjustment, the robot stops; if it is too low, an alert is issued. Dashed lines represent development-phase steps, while solid lines are for both development and deployment phases.

Following the assessment of HRT performance through predictive analytics, as shown in Figure 3, task load management can be achieved by modulating the robot’s operational speed. This action directly influences the task’s temporal demand [32] and indirectly affects other task load indices, thereby altering the overall task load [33]. Such adjustments also modify individuals’ engagement levels with the task. Accurately measuring the task load and developing sophisticated models to predict task load and human performance based on physiological data are essential steps toward guiding individuals to an optimal task load and engagement zone. Implementing these models ensures seamless integration within the human–robot workflow, facilitating an automated HRT system that optimizes performance outcomes.

This study builds upon our earlier research [34], wherein we devised a framework to predict HRT performance through the analysis of task load via eye movement. While our initial findings affirmed that eye movement acts as a proxy for task load and closely correlates with human neurophysiology [29], we encountered limitations, notably that eye movement monitoring is impractical in numerous real-world scenarios. Additionally, our prior work did not offer a methodology to enhance HRT performance following its prediction. To overcome these challenges and expand upon our foundational work, the present study extends our contributions by performing the following:

- Exploring the relationships between task engagement, task load, performance, and physiological data through classification. This framework aims to lay a foundation for enhancing HRT autonomy and optimizing performance.
- Identifying crucial physiological indicators from ML models that correlate with enhanced HRT performance. Analyzing these indicators helps to uncover deeper insights into physiological influences on HRT performance, enabling targeted improvements in team dynamics and efficiency.
- Applying Q-Learning to accurately determine the optimal weights for the NASA TLX questionnaire indices, presenting a versatile and effective approach to assess task load and engagement across diverse tasks and improve human performance.
- Developing a computational pipeline utilizing accessible physiological data to predict and adjust HRT performance in manufacturing environments, aiming to enhance collaboration and productivity and reduce errors.

## 2. Related Work

### 2.1. Task Engagement, Task Load, and Performance

The relationship between task engagement, task load, and performance is a rapidly growing area of research across multiple fields of study. Researchers have focused on analyzing neurophysiological data, such as electroencephalography (EEG), pupilometry, and blood pressure, alongside performance metrics, to evaluate individuals’ levels of engagement when performing specific tasks [16,35]. Another study assessed the effect of task engagement on driving performance. With the increasing availability of sensors and automation systems in modern vehicles, drivers often engage in nondriving tasks while driving, resulting in multitasking. The authors aimed to investigate the impact of the level of engagement employed in parallel activities on drivers’ overall driving performance based on their physiological data [36]. The authors conducted an investigation examining the potential of a concurrent task to improve the outcome of a vigilance task. They used a driving scenario to determine whether a concurrent conversation could enhance a driver’s performance during a monotonous drive, which is typically considered a lower-load task. The study revealed that, for the particular case analyzed, a concurrent conversation was conducive to improving the driver’s vigilance, lane-keeping performance, and steering control [37].

### 2.2. Predicting Task Load Using the NASA TLX

The measurement of human task load is critical to ensure safety and efficiency in various industries, including healthcare. The NASA TLX questionnaire is a commonly used tool to assess task load. In healthcare, tasks with high loads can negatively affect patient safety and increase the risk of stress-related illnesses. Researchers have utilized the NASA TLX to measure the task load of Intensive Care Unit (ICU) nurses and found it to be the most reliable method. Their study provides benchmark data for healthcare managers to evaluate the task load of their ICU nurses [25]. Another study in healthcare sought to assess the task load of surgeons performing laparoscopic procedures in a virtual reality setting. The authors combined eye-tracking data analysis with the NASA TLX questionnaire scores to characterize the task load of the surgeons. By analyzing the eye-tracking data and using the NASA TLX results as a reference, the authors determined that blinking frequency and duration can be used to estimate task load [38]. Previous research has shown the usefulness of the NASA TLX questionnaire in designing machine equipment. In this study, the authors tested two Human–Machine Interface (HMI) designs for excavator operators to determine their impact on physical and mental task load. The NASA TLX questionnaire was used to measure the mental load experienced by the operators while interacting with the different HMI designs. The results indicate that the different HMI designs directly affected the operators’ physical and mental task loads. Based on these findings, the authors identified the optimal HMI design [39].

### 2.3. Physiological Data for Prediction of Task Engagement and Task Load

Previous research has indicated that electrodermal activity, respiratory rate, and accelerometry data, in combination with the Flow Short Scale, a self-reported engagement measure, can be utilized to accurately assess learner engagement levels. These measures have been effective in discriminating between engagement levels across different environments, such as classroom, simulation, and live settings, using physiological data [40]. Also, by monitoring heart rate and electrodermal activity measures, the researchers were able to identify individual differences in electrodermal liability, which predicted whether participants would engage affectively or strategically with the emotionally expressive agents in reacting to emotionally expressive agents during the decision task [41]. Moreover, a supervised ML approach was employed to classify the level of cognitive load based on physiological feedback. Specifically, a random forest algorithm was used, resulting in an accuracy of 94% in decoding the physiological data associated with the stated cognitive load in the NASA TLX [1].

Previous research has predominantly explored the relationship between cognitive load—a singular index of the NASA TLX—and physiological data, or it has utilized physiological measurements to assess task load and engagement. However, a holistic methodology that integrates all indices of task load, engagement, performance, and physiological data to evaluate and subsequently enhance performance in work environments remains unexplored. This study seeks to bridge this gap by delving into the interconnections among these diverse data types, encompassing various physiological signals, task load indices, and HRT performance metrics. It aims to identify the physiological features that most significantly influence task performance. Although electroencephalography (EEG) data have yielded promising insights, their high cost and vulnerability to noise limit their practicality. In response, this study adopts a more cost-effective (approximately USD 1700 for a wristband collecting human physiological data) and less susceptible to noise method. Furthermore, this research culminates in the creation of a model designed to predict and enhance task load management and human engagement during task execution.

## 3. Overview of Our Approach

Our framework introduces an innovative method to forecast and optimize HRT performance by examining the dynamics between task load, task engagement, and physiological activation. This approach posits that task load directly influences task engagement (Figure 2) [1,15], which in turn affects performance [17,42,43]—a key metric we predict using physiological data. Central to our strategy is the adjustment of the robot’s speed, a modification aimed at optimizing task load, thereby influencing task engagement and ultimately improving performance. By ensuring that task load, engagement, and performance are maintained within optimal parameters, our framework seeks to maximize efficacy in QC tasks. To achieve this, we developed a computational pipeline designed to precisely predict HRT performance in QC scenarios, striving for peak performance.

### 3.1. Performance Prediction

As illustrated in Figure 3, performance prediction is achieved by utilizing participants’ QC performance data (Section 5.1) and NASA TLX questionnaire responses to determine the task load experienced post-experiment (Section 5.2). The NASA TLX evaluates task load across six indices, and a weighted average is computed from the responses, which range from 1 to 20 for each index. Q-Learning is applied to determine task-specific weights, critical for assessing the imposed task load on individuals. These weights inform the creation of three performance labels: inaccurate due to disengagement, accurate within optimum engagement, and inaccurate due to stress. Our approach replaces the use of the NASA TLX questionnaire and QC performance records with physiological data, which are continuously recorded during HRT tasks, to predict performance without interrupting the workflow (Section 5.3). Subsequently, we show the effectiveness of our weighted average TLX approach, facilitated by Q-Learning for performance prediction (Section 5.4).

### 3.2. Performance Maximization

For performance maximization, the framework’s objective is to optimize HRT performance through task load adjustments within the HRT workflow, primarily by modulating the robot’s operational speed to adjust the temporal load, as detailed in Section 5.5. Our methodology, depicted in Figure 3 (in the Performance Maximization part), begins by correlating physiological data with temporal load experienced by participants. We then assess the direct and indirect effects of temporal load alterations on task performance through robot speed adjustments. This analysis aims to identify the ideal robot speed adjustment that ensures participants operate within the most favorable task load conditions. If adjustments to the robot’s speed cannot adequately manage task load levels that are too high or too low, the framework recommends implementing rest breaks or providing timely warnings, respectively. These interventions are considered through a monitor placed adjacent to the robot, serving as a channel for visual feedback. The overarching aim is to enhance HRT performance while keeping task load and stress at manageable levels.

## 4. Materials and Methods

### 4.1. Participants

This study involved 22 graduate and undergraduate students, of which 10 were female, with an age range of 19 to 37 years and a standard deviation of 5.3 years. Prior to the experiment, all participants demonstrated proficiency in using QC devices, including the caliper, to ensure that they could perform the task accurately. However, their experiences using calipers and working with robots varied. While some participants had prior experience working with robots on a daily basis, others had limited or no experience working alongside a robot. Additionally, participants were instructed to complete the task as quickly as possible, resulting in variations in their completion time relative to the robot’s pace. Some participants completed the measurements before the robot brought in a new part, while others fell behind and were unable to complete the necessary measurements before the arrival of the next part. These variations in participants provided diverse performance, task load, and physiological data.

### 4.2. Experiment

**Figure 4:**
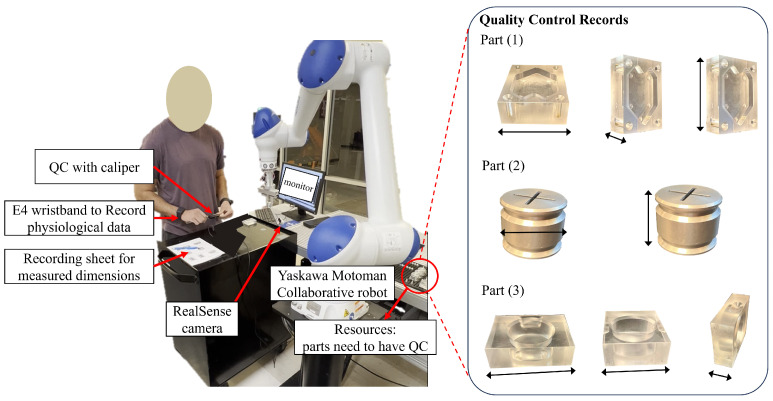
The HRT experiment setup featuring the parts subject to measurement, encompassing 8 distinct dimensions.

In this experiment, which was approved by the Institutional Review Board of The University of Alabama (23-01-6294), a collaborative HRT simulated an industrial application of QC for manufactured parts, as depicted in Figure 4. The experimental setup comprised a measurement station, a caliper as the measurement tool, a HC10DT Yaskawa collaborative robot (Kitakyushu, Japan), an Intel RealSense D455 camera (Santa Clara, CA, USA), two E4 wristbands, and three manufactured parts. The Empatica E4 wristband (Boston, MA, USA) is a noninvasive, wearable research device designed to collect physiological data.

### 4.3. Data Collection

During data collection, the human operator wore an E4 wristband on each wrist, while the robot transported the manufactured parts to the measurement station. As the parts arrived at the measurement station, the human operator performed the assigned measurements using a caliper and recorded the corresponding values on a control document. Each participant measured eight dimensions across three parts during HRT for QC task. The data collection was conducted in two scenarios: 1—Normal Speed Human–Robot Teamwork (NS-HRT) with the robot operating at regular speed (225 mm per second); 2—Reduced Speed Human Detection-Based HRT (RS-HD-HRT), where the robot reduced its speed to one-third of its original speed upon detecting the human operator within the robot’s work envelope. The average completion time of participants for the QC task in the NS-HRT scenario was 2 min and 59 s, and 3 min and 54 s in the RS-HD-HRT scenario. Two different scenarios were designed for data collection to observe the impact of varying robot behaviors (i.e., robot’s speed) on participants’ task load. Therefore, conducting the experiment in two different scenarios helps to collect various data that can contribute to developing a more robust prediction model. Following the collection of the assigned measurements, the participants filled out the NASA TLX questionnaire to assess the task load experienced while performing the QC task alongside the robot.

### 4.4. Data Preprocessing

To facilitate the analysis of the collected data, automated recording of the start and end times of the experiments was performed. The E4 wristband also generates the start time of the recording automatically and the relevant portion of data falling within the experiment time was extracted. The E4 wristband was used to acquire various physiological data, including hand acceleration (ACC), blood volume pulse (BVP), heart rate (HR), electrodermal activity (EDA), and skin temperature (TEMP). The sampling frequencies for these physiological data were 32, 64, 1, 4, and 4 data samples per second, respectively.

## 5. Results

### 5.1. Performance Evaluation

**Figure 5:**
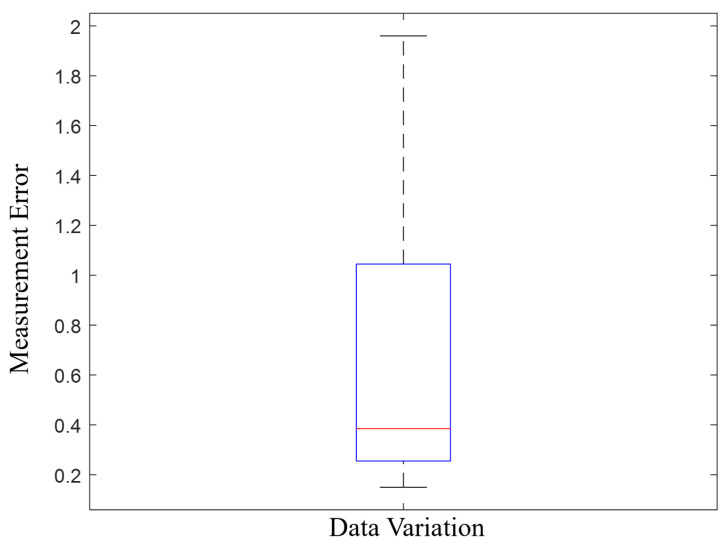
The distribution of the participants’ performance (Box plot of measurement error as a function of data variation. The central red line within the blue box represents the median error value. The edges of the blue box depict the interquartile range, indicating the middle 50% of the data. The dashed lines extending vertically from the box indicate the variability outside the upper and lower quartiles, representing the total spread of the data).

To evaluate the participants’ performance, the absolute values of measurement error (*ME*) were used:

(1) $$\begin{array}{c} ME=\sum_{i=1}^{8}\left|D_{c}^{i}-D_{p}^{i}\right| \end{array}$$

where $D_{c}^{i}$ and $D_{p}^{i}$ represent the correct measurements and the participants’ measurements for the *i*-th part in the quality control (QC) task, respectively. As demonstrated in Figure 4, each participant measured a total of eight dimensions across three different parts in each HRT scenario. The distribution of the ME is depicted in Figure 5. The minimum, maximum, and standard deviation of the ME are 0.15 mm, 1.96 mm, and 0.4895 mm, respectively. The top-performing third of the participants were deemed to have achieved the best performance and were used to determine the weights for the NASA TLX. This method of selecting the highest-performing participants allowed for a more accurate determination of the weighting factors for each of the TLX indices. By focusing on the top performers, we were able to derive a more precise set of weights that reflected the most efficient and effective ways of managing task load levels during the HRT task.

### 5.2. NASA TLX Analysis with Q-Learning

**Figure 6:**
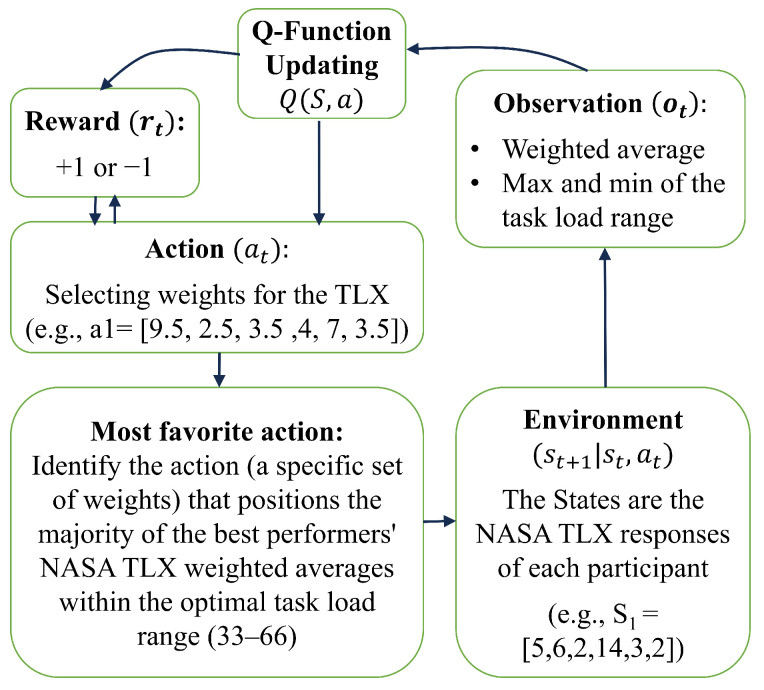
Application of the Q-Learning algorithm to determine the task load weights that place the maximum possible number of participants, with the highest performance, within the optimal range. A reward of +1 is assigned for weighted averages falling within this optimal range, while a reward of −1 is given for those outside of it.

We employed a Q-Learning algorithm, illustrated in Figure 6, to identify the weights that produce a weighted average of the NASA TLX responses from the participants with the best performance. The Q-Learning algorithm utilizes a Q-table, a matrix combining all possible states and actions, to record expected rewards for actions based on given states. The Q-table is initialized with zeros, and initial weights are set to one. These weights are iteratively adjusted. The weighted average TLX score for a given set of weights (an action) is calculated next. To determine the optimum range for task load, we considered a range of 33–66 on a scale of 1–100, which results in the optimum engagement of the participants [1]. The algorithm then assesses the reward for a specific weighted average to ascertain if it falls within this optimum range. An epsilon-greedy policy directs the action selection process in each state. The Q-Learning updates within each episode are conducted as follows:

1. Initiating the process with an initial state that reflects the beginning of a task or specific conditions.
2. Making decisions based on the current policy by selecting weights for the TLX indices.
3. Observing outcomes to assess whether the resulting task load falls within the optimal range of 33–66.
4. Receiving rewards based on the effectiveness of the chosen weights in placing the task load within the optimal range.
5. Updating the policy by refining the Q-values, thereby enhancing the strategy for subsequent decision-making processes.

The fundamental Q-Learning update rule applied in our algorithm is represented by the following equation:

(2) $$Q\left(s,a\right)\leftarrow Q\left(s,a\right)+\alpha \left[R\left(s,a\right)+\gamma \max_{a^{\prime }}Q\left(s^{\prime },a^{\prime }\right)-Q\left(s,a\right)\right]$$

where, Q(s,a) is the current Q-value of being in state *s* and taking action *a*, α is the learning rate, R(s,a) is the reward received after taking action *a* in state *s*, γ is the discount factor, $\max_{a^{\prime }}Q\left(s^{\prime },a^{\prime }\right)$ is the maximum Q-value for the next state s’ over all possible actions a’, and Q(s,a)← denotes the update of the Q-value.

Our Q-Learning model’s states are derived from the NASA TLX questionnaire’s six task load indices, defining six dimensions per state. Actions in this model adjust weights for these indices to compute a weighted average task load, creating an action space with six dimensions as well—one for each task load index. Weighted average values are calculated as shown in Equation (3):

(3) $$\begin{array}{c} \mathrm{weighted}\;\mathrm{average}\;\mathrm{of}\;\mathrm{the}\;\mathrm{TLX}=\frac{\sum_{j=1}^{6}w_{j}TLX_{j}}{\sum_{j=1}^{6}w_{j}}\times 100 \end{array}$$

where $TLX_{j}$ represents the participants’ responses to the NASA TLX indices, and $w_{j}$ are the weights assigned to each of these indices. To optimize computational efficiency, the range of potential action weights was limited to increments of 0.5, extending from 0.5 to 20. This constraint yields a total of $40^{6}$ possible combinations of action weights. We established a reward mechanism: a reward of +1 is granted for any set of weights that positions a participant’s weighted average within the optimal performance range. Conversely, a penalty of −1 is applied for weights resulting in averages outside this range, thus incentivizing the alignment of participant performance with desired outcomes.

The Q-Learning process was conducted over up to 1000 episodes. During each episode, the agent selects actions (*a*) based on the ϵ-greedy policy, as defined in Equation (4). This policy dynamically adjusts between exploring new actions and exploiting known rewards, guided by the decay of ϵ outlined in Equation (5). After executing an action, the agent assesses the reward and updates the Q-values in accordance with Equation (2). This iterative process enables the agent to refine its strategies continually, optimizing the learning of effective policies over time.

In our Q-Learning framework, initially set to 1.0 to foster exploration, ϵ undergoes gradual reduction, as specified by Equation (5), transitioning the agent towards prioritizing more reliable actions as it gains confidence in its learned values. This controlled reduction in ϵ prevents premature convergence on suboptimal policies and promotes thorough exploration of the action space. Table 1 below details the hyperparameters used in our Q-Learning model, including the learning rate (α) and the discount factor (γ), which are essential for influencing how future rewards are valued relative to immediate rewards.

(4) $$a=\left\{\begin{array}{cc} \mathrm{random}\;\mathrm{action} & \mathrm{with}\;\mathrm{probability}\;\epsilon ,\\ \arg\max_{a^{\prime }}Q\left(s,a^{\prime }\right) & \mathrm{with}\;\mathrm{probability}\;1-\epsilon . \end{array}\right.$$

(5) $$\epsilon \leftarrow \max(\epsilon_{\min},\epsilon \times \epsilon_{\mathrm{decay}})$$

**Figure 7:**
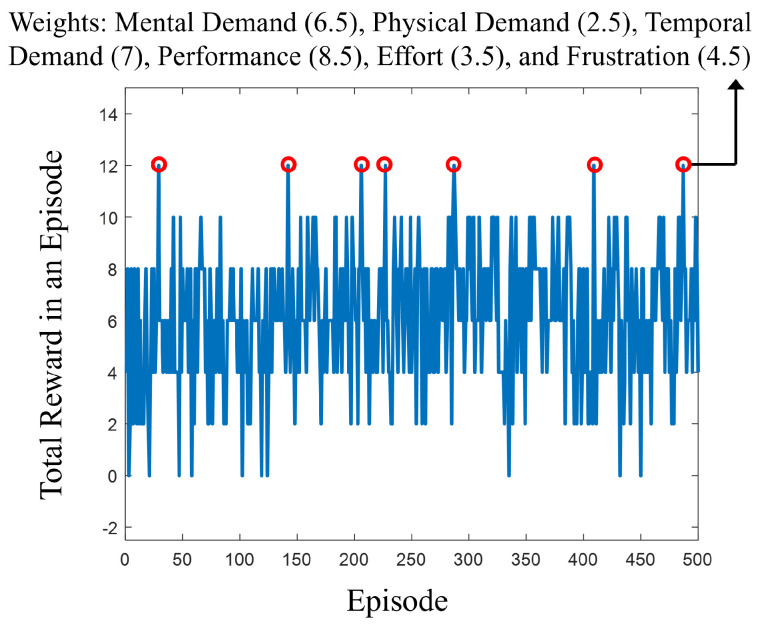
The relationship between rewards and episodes during the training of the Q-Learning algorithm, aimed at identifying the optimal weights. These weights adjust the NASA TLX weighted averages to fall within the optimal range. The highest reward obtained was 12, which is indicated by red circles.

Our analysis determined the optimal weights for the NASA TLX indices to be Mental Demand at 6.5, Physical Demand at 2.5, Temporal Demand at 7, Performance at 8.5, Effort at 3.5, and Frustration at 4.5. This particular set of weights was deemed optimal, as it achieved the highest reward of 12 in our model, as shown in Figure 7. This indicates that the weights, derived from our Q-Learning process, successfully categorized 13 out of 14 participants’ responses within the desired task load range of 33 to 66. While there are a few other weight configurations that reach a reward of 12, they are very close to these identified optimal weights.

**Figure 8:**
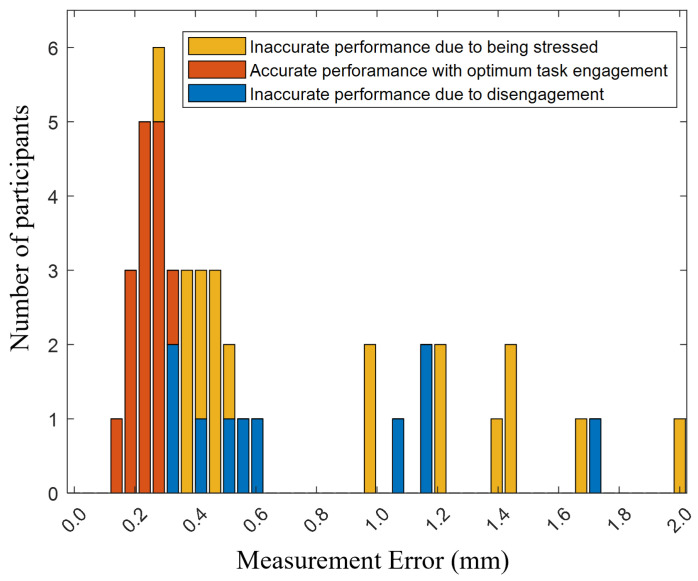
Histogram of measurement errors: This figure illustrates the distribution of measurement errors spanning from 0 to 2 mm in quality control tasks, where task load indices weights, derived through Q-Learning, are applied to categorize participants’ performance and engagement. Each bar indicates the frequency of occurrences within specific measurement error ranges, categorized by performance accuracy: blue for accurate measurements within optimal engagement, orange for inaccuracies due to disengagement, and yellow for errors associated with stress.

After applying the determined weights to the remaining participants’ responses, a weighted average was calculated for each. This process classified 10 responses as inaccurate due to disengagement (below 33) and 19 responses as inaccurate due to stress (above 66), while 15 responses fell within the optimum range (33–66). Notably, the count of responses in the optimum range rose from 13 to 15, attributable to the inclusion of two additional participants’ responses, specifically ranked 15th and 17th in terms of performance, based on calculations with Equation (1). These responses were placed in the optimum range following the application of the weights for weight averaging. In this regard, the histogram distribution of participants ranging from 0.15 to 1.96 in terms of ME, is presented in stacked bar charts in Figure 8. Lower measurement errors indicate higher performance, while higher measurement errors signify lower performance. The vertical axis of the histogram in Figure 8 displays the count of participants grouped by these measurement error intervals.

### 5.3. Task Performance Prediction Using Physiological Data

Maximizing HRT performance without interrupting human–robot collaboration requires predicting HRT performance through a computational pipeline, as shown in Figure 3. The pipeline utilizes information from humans’ stated TLX, performance, and physiological states while working with a collaborative robot. To clarify, the collection of TLX responses and performance evaluation are conducted exclusively during the model/pipeline training phase, after which the model can operate solely on physiological data inputs. Previous sections discuss the performance evaluation, analysis of NASA TLX, determination of optimized Q-Learning weights, and their importance. This section focuses on the prediction of HRT performance using physiological data without disrupting the collaboration.

To establish the relationship between task load and physiological data through an ML model, it is essential to generate high-quality features. The method of obtaining such features is crucial for achieving accurate results. Traditional manual selection methods, such as choosing statistical measures such as mode and mean, have been shown to capture only a fraction of the physiological signal’s complexity. Specifically, characteristics such as nonlinear dynamics, temporal variations, and spectral components, which are crucial for a comprehensive representation of human physiology, are often neglected. This oversight can result in a poor depiction of physiological activities [44]. To surmount these limitations, we employed the tsfresh feature engineering library [45], which systematically and automatically extracts a broad spectrum of statistical features, thus encompassing the intricate nature of physiological signals more effectively.

For each HRT scenario (i.e., NS-HRT and RS-HD-HRT), feature extraction from physiological data was performed, and a total of 1109 features were computed for each type of physiological data (i.e., ACC, BVP, HR, EDA, and TEMP), amounting to a combined 5545 features. The feature space was comprehensive, leveraging all available statistical features provided by tsfresh, which includes simple features such as mean, as well as more advanced ones, such as entropy of the power spectral density, thereby ensuring a nuanced analysis of the physiological data.

However, the extensive feature set presented challenges related to computational efficiency and the potential for model underperformance due to irrelevant feature inclusion. To address these concerns, we streamlined the feature set by implementing a feature significance testing procedure postextraction. Utilizing the one-way analysis of variance (ANOVA) F-value enabled us to discern the most predictive features, narrowing the focus to the top 200 features for subsequent classification tasks. This approach not only mitigated computational burdens but also enhanced model accuracy by prioritizing features with the highest relevance to the target outcome. The efficacy of this refined feature set is demonstrated in the classification results presented in Table 2.

In this study, six models were developed for predicting the engagement level of individuals during HRT tasks. These models include Naive Bayes, K-Nearest Neighbors (KNN), Decision Tree (DT), Linear Discriminant (LD), Neural Network (NN), and Support Vector Machine (SVM). Accurate performance with optimum engagement, inaccurate performance due to disengagement, and inaccurate performance due to stress are the three classes of ground truth for the predictive models. In this regard, 70% of the data were utilized for training, 15% for validation, and 15% for testing. To prevent the ML model from exhibiting bias towards any specific data subset, it is important to conduct a fair evaluation. Therefore, a 5-fold cross-validation approach was utilized to obtain the results from the average of all folds. The evaluation of the model’s performance was based on five criteria, which include accuracy, kappa, recall, precision, and the Area Under the Curve (AUC) of the Receiver Operating Characteristic (ROC). These metrics were used to evaluate the performance of the models on the test data. The accuracy represents the percentage of correctly classified samples. The kappa measures the agreement between the predicted and actual labels. The recall calculates the proportion of true positives over the total actual positives, while the precision measures the proportion of true positives over the total predicted positives. Additionally, the average AUC for all classes indicates the probability of ranking a random positive example higher than a random negative example.

Moreover, our analysis of feature importance revealed that certain features used in ML played a critical role in predicting human performance. Specifically, the ACC Fast Fourier Transform (FFT) features, HR Fourier entropy, and EDA number of peaks were found to be the top three important features. These features can potentially provide valuable insights into the underlying physiological processes that affect human performance during QC tasks in HRT.

### 5.4. Results without Using Q-Learning in TLX Weight Determination

**Figure 9:**
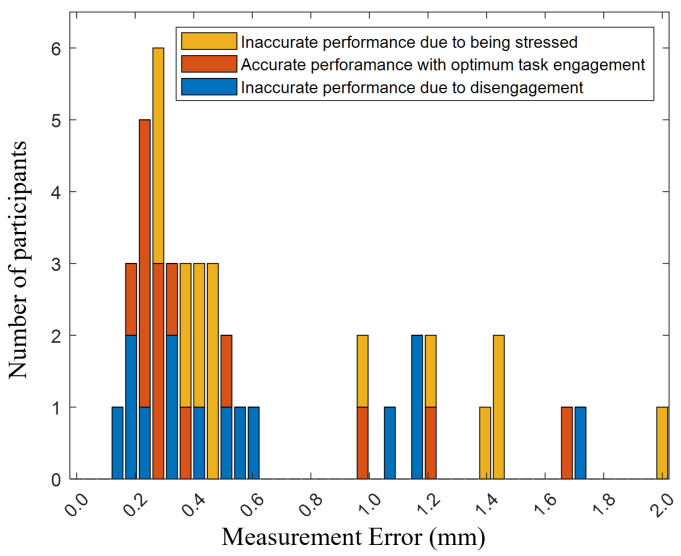
Histogram of measurement errors: This figure illustrates the distribution of measurement errors spanning from 0 to 2 mm in quality control tasks prior to applying weighted task load indices. Each bar represents the frequency of errors within specific measurement ranges, categorized by performance accuracy: blue for precise measurements within optimal engagement, orange for inaccuracies due to disengagement, and yellow for errors induced by stress.

To further clarify the role of weighted task load indices obtained with Q-Learning in the developed pipeline for predicting the performance of HRT, the results without using Q-Learning to find the optimal TLX weights are presented. In this case, normal averaging is employed, and each index of the TLX is assigned a weight of one. The resulting performance distribution of the participants, which is calculated with Equation (1), is shown in Figure 9. As depicted in the figure, due to the inappropriate weight assignment to the TLX indices, an acceptable distribution between task engagement and performance cannot be observed.

Furthermore, the ML approaches employed, as demonstrated in Table 3, cannot generate a dependable prediction based on the labels obtained through normal averaging to determine task engagement. Thus, the proposed framework with using Q-Learning can play a significant role in predicting the performance of HRT in a QC task, and the developed computational pipeline could be utilized for any other task to forecast task performance based on physiological data.

### 5.5. Utilizing Q-Learning for Task Load Optimization and Maximization of HRT Performance

In this section, we aim to take actions that optimize the task load and enhance HRT performance by adjusting the HRT task load to improve human engagement and maximize their productivity. The primary adjustable parameter within our control is the robot’s speed. Upon predicting HRT performance using ML on physiological data, it is determined whether the human is disengaged or stressed, as depicted in Figure 2 in the Performance Maximization part. Altering the robot’s speed has a direct influence on the temporal demand aspect of the NASA TLX and indirectly impacts the other five indices. These changes, both direct and indirect, affect the overall task load, subsequently influencing human engagement and performance. We employ Q-Learning to ascertain the optimal adjustments to the robot’s speed (i.e., the temporal demand) to enhance HRT performance, taking into account the consequent effects on all indices of the NASA TLX. Figure 3 illustrates the comprehensive framework that integrates the prediction of HRT performance based on ML analysis of physiological data with task load adjustments, thereby creating a feedback loop aimed at optimizing HRT performance.

#### 5.5.1. The Direct Impact of Altering Temporal Demand on Task Load

**Figure 10:**
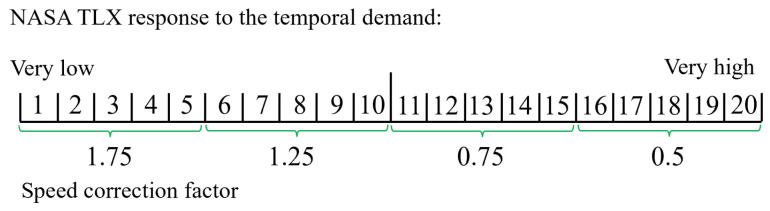
The division of four temporal sections and corresponding speed correction factor to apply to the robot’s speed to modify the human task load and thus modify the human’s engagement.

In order to maintain an uninterrupted workflow during the HRT experiment and ensure full automation, it is essential to identify the physiological data that correspond to the temporal demand index of the task load. Once the predicted performance is inaccurate due to stress or disengagement, the temporal demand, i.e., the robot’s speed, needs to be adjusted by a factor to keep the human in the optimum task load range. However, accurately classifying physiological data for each of the 20 temporal demand values of NASA TLX is not feasible, so we divided the temporal demand into four intervals. ML is employed to achieve the classification of the four temporal demand sections using the physiological data features explained in Section 5.3, and the results are presented in Table 4, in which the best performance of 94.32% is achieved by the Decision Tree method. The next step involves assigning a speed correction factor to each of the four temporal demand classes to adjust the temporal demand and, consequently, the overall task load. In the NASA TLX, a response of 1 is deemed very low, and a response of 20 is deemed very high. The temporal classes and corresponding speed correction factors are illustrated in Figure 10, where to address the nonoptimal engagement, the robot’s speed can be modified by these factors. When the temporal demand is low, increasing the robot’s speed can raise both the temporal demand and the overall task load, potentially improving task engagement and so performance. Conversely, when temporal demand is high, reducing the robot’s speed may lower the temporal demand and overall task load, thereby moving engagement closer to the optimum range and thus improving performance.

#### 5.5.2. The Indirect Impact of Temporal Demand Alterations

When the temporal demand (i.e., the speed of the robot) is modified, it indirectly impacts the other indices of the TLX. To determine the effects, we calculated the correlation coefficient between the change in the temporal demand and the Mental Demand, Physical Demand, Expected Performance, Effort, and Frustration indices of the TLX. To this end, a vector of the temporal demands in the NASA TLX a vector of another NASA TLX index (e.g., Mental Demand) was input to the corrcoef function in MATLAB R2023b. The obtained correlation coefficients were 0.6684, 0.7929, 0.6749, 0.5722, and 0.7481, respectively. The positive correlation observed between the temporal demand index of the NASA TLX and the other indices implies that an increase in temporal demand (i.e., robot speed) leads to higher levels of mental and physical demand, a greater sense of task failure, increased effort required to accomplish the task, and more negative feelings such as stress. Thus, changing the temporal demand (i.e., the robot’s speed) can affect the overall task load. For instance, if the temporal demand (i.e., robot speed) needs to be adjusted by a factor of 1.75 to optimize the overall task load, then the Mental Demand, Physical Demand, Expected Performance of the participants, the Effort required to complete the task, and their stress levels are expected to change by factors of 0.6684×1.75, 0.7929×1.75, 0.6749×1.75, 0.5722×1.75, and 0.7481×1.75, respectively.

#### 5.5.3. Q-Learning for Task Load and Performance Adjustment in HRT

**Figure 11:**
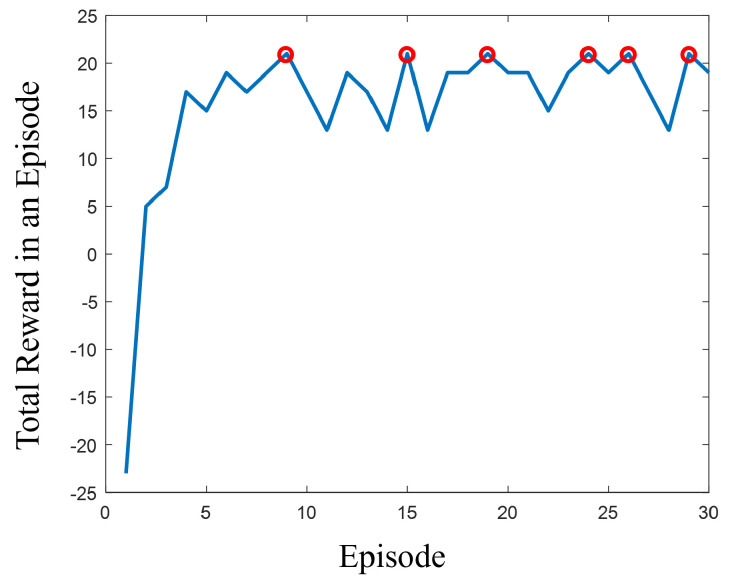
Reward per episode across Q-Learning training sessions aimed at optimizing temporal demand to enhance HRT performance. Peak rewards of 21, indicated by red circles, signify successful task load adjustments for 25 out of 29 participants, previously underperforming due to stress or disengagement, to an optimal range.

In this section, we developed a Q-Learning method to fine-tune the task load of the participants who had low performance in the HRT QC task, leveraging participants’ NASA TLX questionnaire responses as states, similar to the process described in Section 5.2. The action space, similar to that in Section 5.2, consisted of weights assigned to task load indices. However, the inclusion of robot speed correction factors (Figure 10) was also applied to evaluate task load adjustments resulting from changes in robot speed. Rewards were assigned a value of +1 when adjustments to the robot’s speed—impacting both the temporal demand index directly and other TLX indices indirectly—brought the weighted TLX average within the optimal range of 33 to 66 on a 100-percent scale. Conversely, a reward of −1 was applied when the adjustment failed to achieve this range. The observation space encompassed the new weighted averages following temporal demand adjustments. Utilizing this reward system, we produced a graph of rewards per episode (Figure 11), demonstrating that speed modifications successfully optimized the task load for 25 out of 29 participants previously showing inaccurate performance due to stress or disengagement, thus enhancing their performance.

To avoid relying on performance records and the NASA TLX during HRT operations, we trained ML algorithms to classify participants’ physiological data, particularly those showing low performance, across four temporal demand intervals. This classification, detailed in Table 4, achieved its highest accuracy at 94.32% using the DT ML model. Therefore, in HRT scenarios, after identifying low-performance instances in QC tasks through physiological data (as explained in Section 5.3), the algorithm predicts which temporal demand interval the participant’s physiological data falls into. Subsequently, corresponding robot speed correction factors for each interval (Figure 10) are applied to adjust the task load, thereby enhancing participant engagement and performance in QC tasks without disrupting the HRT workflow.

## 6. Discussion

In this paper, we presented a novel framework with Q-Learning designed to fine-tune task loads in HRT during QC tasks, utilizing physiological data and task load indices to predict and improve HRT performance. The rationale behind categorizing performance inaccuracies according to their root causes—namely task load and engagement—is to pinpoint effective strategies for enhancing performance. One such strategy involves adjusting the robot’s speed to better align human engagement with the task at hand, thereby optimizing the HRT workflow. This approach enables our framework to adapt dynamically to varying task loads and levels of human engagement, directly influencing and potentially enhancing performance outcomes.

Our proposed framework is a significant step forward in the domain of adaptive Human–Machine Interaction, as it provides the ability to dynamically adjust task parameters in response to human physiology could lead to more responsive and efficient HMI environments, potentially reducing error rates and improving overall productivity. This novel approach can have implications in different sectors.

In the automotive industry, this innovative approach enables the adjustment of automobile speeds and operational parameters, based on the driver’s engagement level and workload as indicated by their physiological signals. Such a system could significantly enhance driving safety and efficiency by automatically modulating vehicle dynamics to align with the driver’s current state, ensuring optimal alertness and responsiveness. For instance, if physiological data suggest that the driver is becoming fatigued or less attentive, the vehicle could automatically adjust its speed or provide alerts to encourage a break. This method goes beyond conventional safety features by creating a more intuitive interaction between the vehicle and its operator, promising to reduce the likelihood of accidents and improve the driving experience in scenarios ranging from daily commutes to long-haul journeys.

The healthcare sector can benefit from our approach by enhancing the performance of staff in high-stress environments, such as surgery or emergency care. By monitoring physiological indicators of stress and engagement, our framework can adjust task loads in real time, potentially decreasing burnout rates and improving patient care quality. For example, in robotic-assisted surgeries, adjusting the operational parameters of robotic systems in response to the surgeon’s stress levels could lead to more precise and safer surgical outcomes.

Given potential concerns that our study may appear overly concentrated on a specific simulated QC task, we aim to underscore the expansive applicability of our framework across various HRT environments. Although our current application predominantly adjusts the speed of a robotic arm to modulate task load, the foundational principles of our approach are designed with the flexibility to adapt to a wide range of operational parameters and distinct robotic systems. For instance, in surgical robotics, the modulation of robot autonomy levels is pivotal. Adjusting the autonomy based on the surgeon’s real-time cognitive load can significantly enhance both performance and safety. Similarly, within assembly line operations, dynamically altering the duration of pauses between tasks can effectively mitigate operator fatigue, thereby enhancing productivity and operational efficiency.

In logistics and warehousing, where timely and accurate processing of goods is critical, our approach can optimize the performance of workers interacting with robotic systems for inventory management and order fulfillment. By ensuring that human operators are neither overburdened nor underengaged, our system can contribute to smoother operations, reduce workplace accidents, and improve overall throughput.

Our feature importance analysis, identifying key physiological features such as ACC Fast Fourier Transform, HR Fourier entropy, and EDA number of peaks, is pivotal for predicting HRT performance and offers valuable insights for further human behavior research. It provides quantifiable data on how various activities, environments, or stimuli impact human physiological responses, potentially unlocking new understanding in fields such as psychology, cognitive science, and social sciences regarding human emotions, stress responses, and engagement. Moreover, these critical features play a vital role in evolving our framework towards real-time application. By pinpointing these markers as essential, we significantly streamline the necessary feature set for precise performance prediction. This streamlining is crucial for developing a real-time version of our framework, facilitating efficient data processing and faster decision making. Implementing such optimized, adaptive systems in real-world settings can markedly improve interaction quality and the efficacy of HRT systems, underscoring the practical benefits of our findings and their contribution to enhancing real-time human–machine interaction technologies.

While our research introduces a promising approach for predicting and enhancing HRT performance, it faces certain limitations. A key constraint is our inability to integrate the collected data directly into our model for real-time application in HRT tasks, primarily because the available wristbands used for data collection cannot transmit data in real time but only after recording is complete. This limitation impacts the potential for immediate data analysis and application. Moreover, utilizing real-time data poses challenges to the accuracy of machine learning models due to the dynamic nature of live data streams. Another challenge is our reliance on high-quality, real-time physiological data acquisition, which might not be practical or achievable in various industrial settings. The ML models’ dependence on specific task load indices, task performance records for QC tasks, and Q-Learning strategies also limits its broad applicability and scalability across different tasks or diverse participant groups. Currently, the framework’s effectiveness is validated for only 22 participants and a specific QC task. Future studies are essential to test, adapt, and refine our model for broader contexts and diverse applications.

In addition, in our HRT framework, we have chosen to classify task loads into three distinct categories— inaccurate performance due to disengagement, accurate performance with optimum task engagement, and inaccurate performance due to being stressed—to balance simplicity and practicality in real-time applications, facilitating quick decision-making and implementation of automated responses. While this categorical model has proven effective, achieving a high accuracy of 95.45%, we recognize its limitations in capturing the full spectrum of task load variations. Exploring a continuous model could offer finer granularity and more precise adjustments but would require a significantly larger dataset and also advanced analytical capabilities for real-time integration and processing. Advancing towards such a model is a promising direction for future research, necessitating expanded data collection across diverse HRT scenarios and the development of sophisticated machine learning techniques to manage the complexities of a continuous variable approach. This would enhance our understanding of human–robot dynamics and potentially improve the effectiveness of HRT systems.

Although Q-learning was selected for our initial research, we also considered alternative methods such as SARSA and Deep Q-Networks (DQNs). SARSA, an on-policy algorithm, and DQNs, which use deep Neural Networks to manage high-dimensional state spaces, provide different policy-learning approaches. Q-Learning was preferred due to its computational efficiency, simplicity in implementation, and suitability for scenarios with a low sample size relative to the number of dimensions. Future studies will compare these methods to evaluate their effectiveness in real-world HRT scenarios. Additionally, we plan to explore policy-gradient methods, ideal for continuous action spaces, to potentially enhance interaction quality and responsiveness in HRT systems.

Psychological factors also play a critical role in optimizing HRT, aligning with recent studies that highlight the influence of dynamics from human–human interactions [46]. Factors such as the physical presence of robots, their motor actions, and shared task representations can significantly impact collaborative performance. Inspired by the suggestion to utilize real-time EEG data for evaluating and adapting HRT, our future work aims to integrate biometric feedback mechanisms. This approach will not only enhance the responsiveness of HRT systems but also deepen our understanding of underlying cognitive processes. Implementing advanced techniques that address these psychological and physiological aspects will refine interaction quality and underscore the importance of a multidisciplinary approach in HRT research.

## 7. Conclusions

In today’s manufacturing sector, driven by the advancements of Industry 4.0 and 5.0, there is a significant emphasis on customization, flexibility, and preserving the human-in-the-loop within production workflows. However, managing task load and maintaining engagement levels present challenges, as both excessive and insufficient task loads can result in stress and disengagement, adversely affecting performance in HRT settings. It is crucial to accurately consider, predict, and adjust the task load to optimize the HRT performance without disturbing the workflow. Our study presents a comprehensive framework that predicts HRT performance with a high accuracy rate of 95.45% using a Linear Discriminant Analysis model and fine-tunes task load for higher efficacy. We demonstrate how variations in the robot’s speed can influence key components of task load, including mental and physical demands, performance expectations, effort, and frustration, according to the NASA TLX. This research highlights the value of using physiological data combined with machine learning techniques to improve HRT performance, offering insights into how physiological responses impact human performance during tasks. Notably, our analysis points to the significance of Accelerometer Fast Fourier Transform, Heart Rate Fourier entropy, and Electrodermal Activity number of peaks as crucial predictors of HRT performance, indicating the potential of these markers in enhancing human–robot collaboration.

## Figures and Tables

**Table 1:** Q-Learning Parameters.

Parameter	Value
Exploration Rate (ϵ)	1.0
Minimum Threshold ($\epsilon_{\min}$)	0.8
Decay Rate ($\epsilon_{\mathrm{decay}}$)	0.999
Learning Rate (α)	0.1
Discount Factor (γ)	0.99

**Table 2:** Evaluating classification models utilizing features extracted from physiological data for predicting performance categories obtained with the weighted TLX—accurate performance, inaccurate performance due to disengagement, and inaccurate performance due to stress. The maximum accuracy, 95.45%, is achieved by the LD method.

Model	Accuracy (%)	Kappa	Recall (Weighted Mean in %)	Precision (Weighted Mean in %)	AUC
Naive Bayes	82.95	0.6165	82.05	81.45	0.8941
KNN	81.82	0.5909	77.37	82.02	0.9129
DT	86.36	0.6932	85.70	87.31	0.9029
LD	**95.45**	0.8977	94.44	95.00	0.9353
NN	92.94	0.8412	92.78	92.82	0.9941
SVM	94.32	0.8722	93.57	93.44	0.9989

**Table 3:** Evaluating classification models utilizing features extracted from physiological data features to classify three performance categories—accurate performance, inaccurate performance due to disengagement, and inaccurate performance due to stress—without incorporating Q-Learning-derived labels. The highest accuracy of 64.77% is achieved by the KNN method.

Model	Accuracy (%)	Kappa	Recall (Weighted Mean in %)	Precision (Weighted Mean in %)	AUC
Naive Bayes	57.95	0.0540	57.70	57.57	0.6923
KNN	**64.77**	0.2074	65.21	64.88	0.7405
DT	63.64	0.1818	62.91	65.76	0.7024
LD	54.55	0.0222	53.95	54.50	0.6255
NN	61.36	0.1307	60.95	61.01	0.7573
SVM	60.23	0.1051	60.27	60.39	0.7141

**Table 4:** Predicting the temporal demand. The highest accuracy of 94.32% is achieved by the DT method.

Model	Accuracy (%)	Kappa	Recall (Weighted Mean in %)	Precision (Weighted Mean in %)	AUC
Naive Bayes	89.77	0.6804	90.33	91.08	0.9642
KNN	85.23	0.5384	86.21	86.69	0.8836
DT	**94.32**	0.8224	94.17	94.89	0.9952
LD	90.91	0.7159	91.30	90.92	0.9373
NN	93.18	0.7869	93.46	93.28	0.9783
SVM	88.64	0.6449	88.70	90.09	0.9810

## Data Availability

The data presented in this study are available on request from the corresponding author.
